# Bronchial oncocytoma

**DOI:** 10.1590/S1516-31802000000600009

**Published:** 2000-11-01

**Authors:** Renata Telles Rudge de Aquino, Maria Elisa Ruffolo Magliari, Roberto Saad, Vicente Dorgan, Jorge Ethel, Carlos Dandretta, Vera Luiza Capelozzi

**Keywords:** Oncocytoma, Bronchial, Pulmonary, Oncocitoma, Brônquico, Pulmonar

## Abstract

**CONTEXT:**

Oncocytomas are generally small and present slow growth. Finding of the tumor usually occurs incidentally. Their incidence is higher among male patients. Oncocytomas in mucous bronchial glands are extremely rare.

**CASE REPORT:**

A 35-year-old male who presented bronchial oncocytoma. The tumor was found after bronchoscopy that investigated an atelectasis of the upper left lobe. Histological examination with optical microscopy revealed a mature neoplasm formed by ovoid cells with thin, granular, eosinophilic cytoplasm and small nuclei similar to oncocytes. Electron microscopy showed mitochondrial hyperplasia. A three-year follow-up after thoracotomy followed by lobectomy and removal of the bronchial tumor was uneventful.

## RESUMO

**CONTEXTO:**

Oncocitomas são tumores geralmente pequenos e apresentam crescimento lento. A identificação do tumor usualmente ocorre incidentemente. Sua incidência é maior entre os homens. Oncocitomas originários das glândulas mucosas dos brônquios são muito raros.

**RELATO DE CASO:**

Um paciente de 35 anos do sexo masculino com oncocitoma brônquico. O tumor foi observado à broncoscopia, realizada para investigação de atelectasia do lobo superior esquerdo. O exame histológico com microscopia ótica mostrou neoplasia madura formada por células ovóides com citoplasma eosinofílico fino e granular, com núcleo pequeno, assemelhando-se a oncocitos. A microscopia eletrônica mostrou hiperplasia mitocondrial. O seguimento três anos após a toracotomia com lobectomia e remoção do tumor foi livre de complicações ou recidiva.

## INTRODUCTION

Oncocytomas are rare, usually benign tumors formed by altered epithelial glandular cells named oncocytes. These cells, which were identified by Hamperl in 1931, present abundant eosinophilic cytoplasm, composed of hyperplastic mitochondria and no organelles.^1^

A small number of these cells may be found in the epithelium of the upper respiratory tract and in mucous glands of bronchi and other organs. Their incidence increases with age, although their function remains unknown. Since cell division is not prevented by oncocyte formation, it is possible that they trigger benign or malignant hyperplasias and neoplasms. They may be found in tumors as isolated cells playing a relevant role or as their sole component.^1-3^

Jaffé introduced the term oncocytoma in 1932 for salivary gland tumors formed exclusively or mainly by oncocytes. This designation has been used ever since, although names such as oxyphilic adenoma and mitochondrioma have been suggested. These tumors have been found later in other glandular tissues, such as: thyroid, parathyroid, lacrimal, adenohypophysis, kidneys, and pancreas, all with very similar features regardless of the site.^1,2-4^

Oncocytomas are generally small and present slow growth. Finding of the tumor usually occurs incidentally.^5^ Their incidence is higher among male patients. Oncocytomas in mucous bronchial glands are extremely rare. Fechner and Bentick^6^ reported the first case diagnosed after electron microscopy in 1973. Only nine cases have been reported since then.^2,5,7,8^

## CASE REPORT

**Figure 1:**
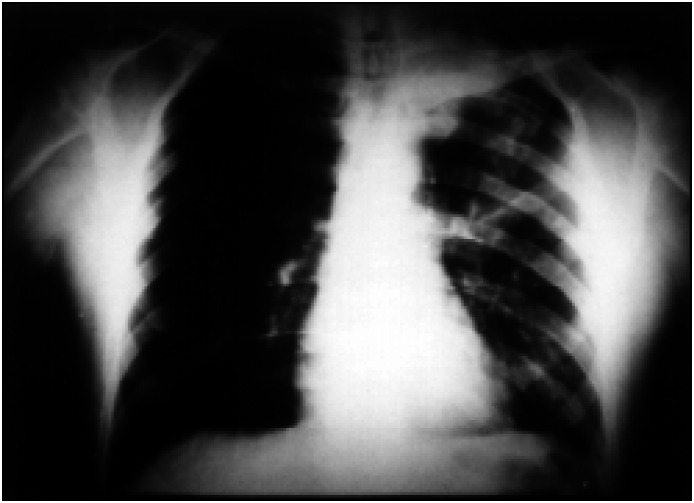
Chest X-ray revealing left upper lobe atelectasis.

**Figure 2:**
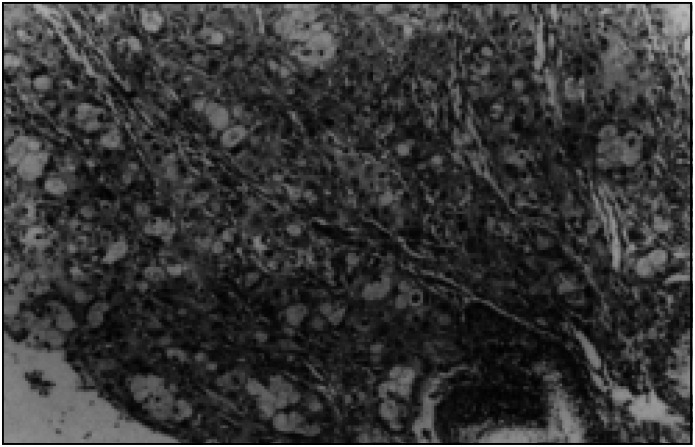
Bronchial section showing proliferation of cells with oncocyte features: well-differentiated uniform cells with granular cytoplasm (HE, x100).

**Figure 3:**
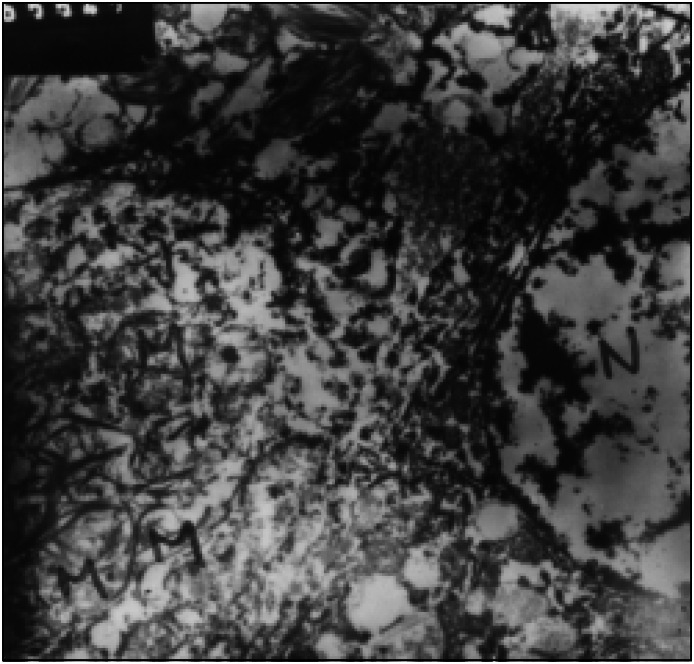
Micrograph showing nucleus with more intense electrondensity close to nuclear membrane. In the cytoplasm we see many mitochondria, poorly preserved by fixation (formaldehyde), evident mainly by mitochondrial cristae (Philips, x9500).

A 35-year-old Hispanic male presented a history of coughing and fever for one week. Physical examination and chest X-rays (Figure 1) revealed left upper lobe atelectasis. Bronchoscopy showed a growth in the upper left lobe. Biopsy of the matter presented a chronic inflammatory process. Computed tomography was not able to detect the tumor. He underwent thoracotomy, lobectomy and removal of the bronchial tumor. His three-year follow-up was uneventful. The findings of the pathology specimens were: a) *Gross Anatomy:* The nodule, with a 1.5 cm diameter, had a smooth surface. Cross-sections showed a yellowtan firm tissue. b) *Microscopic Examination:* Multiple sections, stained with hematoxylin- eosin, showed a mature neoplasm formed by ovoid cells with thin, granular, eosinophilic cytoplasm and small nuclei, similar to oncocytes. In some fields irregular glandular patterns were observed. A fibrous layer, with a small area of bronchial mucosa (Figure 2), limited the nodule. c) *Electron Microscopy:* Ovoid cells with mitochondrial hyperplasia and evident cristae (Figure 3 - Philips - x9500).

## DISCUSSION

Diagnosis was reached after electron microscopy, which revealed mitochondrial hyperplasia. Many organelles may determine granular, eosinophilic cytoplasm, which makes the structural aspects observed in electron microscopy of mitochondrial hyperplasia an important diagnostic criterion.^6^

This case is similar to other reported cases of bronchial oncocytoma in which a tumor was found in a main bronchus of an adult male patient.^2^ The main concern about oncocytomas is their variable malignancy potential. Although usually benign, there are reports of malignant oncocytomas in salivary glands, thyroids, nasal cavities, paranasal sinuses, and the mediastinum.^2^ Nielsen,^7^ in 1985, reported a case of malignant bronchial oncocytoma with lymph node metastasis, whose follow-up after two years of surgical treatment was uneventful. We did not find any other case of bronchial malignancy in the literature.

The biological behavior of pulmonary oncocytomas has not yet been established, due to its rarity. The distinction between hyperplasia, adenoma and carcinoma may be difficult: papillary oncocytomas of the nasopharynx and larynx are usually considered hyperplasias, and the solid lesions arising in salivary glands are considered adenomas. The need for further studies of this rare entity demands the publishing of case reports. In this report, atypical cells, invasion or metastases were not observed; therefore, the tumor was classified as an adenoma with a benign behavior, remaining similar to other reported cases.
